# Propagation of RML Prions in Mice Expressing PrP Devoid of GPI Anchor Leads to Formation of a Novel, Stable Prion Strain

**DOI:** 10.1371/journal.ppat.1002746

**Published:** 2012-06-07

**Authors:** Sukhvir Paul Mahal, Joseph Jablonski, Irena Suponitsky-Kroyter, Anja Maria Oelschlegel, Maria Eugenia Herva, Michael Oldstone, Charles Weissmann

**Affiliations:** 1 Department of Infectology, Scripps Florida, Jupiter, Florida, United States of America; 2 Department of Immunology and Microbial Science, The Scripps Research Institute, La Jolla, California, United States of America; Dartmouth Medical School, USA, United States of America

## Abstract

PrP^C^, a host protein which in prion-infected animals is converted to PrP^Sc^, is linked to the cell membrane by a GPI anchor. Mice expressing PrP^C^ without GPI anchor (tgGPI^-^ mice), are susceptible to prion infection but accumulate anchorless PrP^Sc^ extra-, rather than intracellularly. We investigated whether tgGPI^−^ mice could faithfully propagate prion strains despite the deviant structure and location of anchorless PrP^Sc^. We found that RML and ME7, but not 22L prions propagated in tgGPI^−^ brain developed novel cell tropisms, as determined by the Cell Panel Assay (CPA). Surprisingly, the levels of proteinase K-resistant PrP^Sc^ (PrP^res^) in RML- or ME7-infected tgGPI^−^ brain were 25–50 times higher than in wild-type brain. When returned to wild-type brain, ME7 prions recovered their original properties, however RML prions had given rise to a novel prion strain, designated SFL, which remained unchanged even after three passages in wild-type mice. Because both RML PrP^Sc^ and SFL PrP^Sc^ are stably propagated in wild-type mice we propose that the two conformations are separated by a high activation energy barrier which is abrogated in tgGPI^−^ mice.

## Author Summary

The agent causing transmissible spongiform encephalopathies, the prion, consists mainly if not entirely of PrP^Sc^, an aggregated conformational variant of PrP^C^. PrP^C^ is a host-specified glycoprotein which is attached to the outer surface of the plasma membrane by a glycosylphosphatidylinositol (GPI) anchor. PrP^Sc^ is thought to propagate by seeded conversion, in which PrP^C^ accretes to an aggregate of PrP^Sc^ and thereby assumes its conformation. Prions occur as distinct strains, which have the same PrP sequence but different PrP^Sc^ conformations. Many strains are very stable but some can “mutate”, especially after transient propagation in another animal species. Here “mutation” is attributed to a conformational change of the underlying PrP^Sc^ protein rather than a change in the nucleotide sequence of a gene. In transgenic mice that express anchorless PrP, conversion to PrP^Sc^ occurs extracellularly rather than in a cell-associated compartment. Surprisingly, RML, a very stable prion strain, when propagated in tgGPI^−^ mice and then returned to wild-type mice, was recovered as a novel strain, designated SFL, which was stable over many transmissions. Thus, faithful strain propagation was abrogated either as a consequence of the modified PrP or because of a different replication mode in the extracellular compartment of tgGPI^−^ brain.

## Introduction

Prions, the causative agents of transmissible spongiform encephalopathies, contain as their main component PrP^Sc^, a multimeric conformer of the ubiquitous host protein PrP^C^. PrP^C^ can carry one or two N-linked glycans, whose structure is highly variable [1]–[3], or remain unglycosylated; moreover the glycosylation state varies in different tissues and cell lines, and even in different brain regions [4]–[6]. Most PrP^C^ is attached to the outer surface of the plasma membrane by a glycosylphosphatidylinositol (GPI) anchor [7], but transmembrane forms have been identified [8], [9]. In prion-infected cells PrP^C^ is converted to PrP^Sc^ at the membrane [10] and within the endosomal-lysosomal compartment [11], and accumulates mainly in intracellular compartments [12], [13]. While PrP^C^ is readily cleaved off the cell surface by PIPLC [7], PrP^res^ is not, even though it retains its GPI anchor [14]. PrP^Sc^ presents as a partially proteinase K (PK)-resistant form, designated rPrP^Sc^ or PrP^res^, or as a PK-sensitive entity, sPrP^Sc^ or PrP^sen^ [15]–[17].

Mouse prions occur in the form of a wide variety of strains, which have the same PrP sequence but differ in their incubation time in various mouse lines and in the pattern of lesions they cause in brain [18]. It is believed that strain-ness is encoded by the conformation of PrP^Sc^ and in many instances there are differences in the physicochemical properties of the cognate PrP^res^ [19]–[24]. Interestingly, different prion strains populate distinct regions of the brain [25]–[27] and are selective in regard to the cultured cell lines they can chronically infect [28]–[33]. This raises the unanswered question as to which cellular property underlies prion strain tropism.

The seeding or nucleation model for prion propagation [34]–[36] predicates that PrP^C^ monomers add to PrP^Sc^ and in doing so assume the conformation of its subunits, thus allowing faithful propagation of strains. A prerequisite for chronic infection is that PrP^C^ monomers with a conformation allowing efficient accretion be present at a concentration sufficient to allow synthesis of PrP^Sc^ at a rate higher than that of its depletion [37]–[39]. PrP^C^ is conformationally very flexible [40]–[42] but certain conformations may be more frequent in some cell types but less so or absent in others, which could account for the tropism of prion strains. This proposal begs the question as to why certain conformations would be favored in some cells but not in others. One possibility is that PrP^C^ conformation is modulated by the association of PrP^C^ with a cell-specific component, RNA and phospholipids being two possibilities among several [43], [44]. PrP has a strong binding affinity for polyanions, in particular RNA and homoribopolynucleotides [45], [46]. Binding of RNA causes conformational changes of PrP [47], [48] and *in vitro* conversion of purified PrP^C^ or recombinant PrP to infectious PrP^Sc^ by Protein Misfolding Cyclic Amplification (PMCA) requires the presence of a polyanion and/or phospholipid [44], [49]–[51]. Another possibility is that the highly variable glycosylation of PrP^C^ observed in different cells and tissues [52] might affect its susceptibility to conversion to PrP^Sc^, because of an effect on its conformation [53] or its interactions with putative auxiliary host proteins such as the conjectured protein X [54]. Distinct PrP^C^ glycoforms promote PMCA-mediated prion formation in a species-specific manner [55]. There have been many attempts to explore the role of glycans in the fidelity of strain propagation, in particular by generating amino acid replacements at the glycosylation sites [53], [56]–[58]. However, in these experiments failure to propagate prions or to propagate them faithfully could be due to sequence changes, which are known to affect efficiency of propagation and cause strain shifts [59], rather than the lack of glycans. We were therefore interested in investigating the effect of post-translational modifications of PrP^C^ on the fidelity of strain propagation in the absence of an amino acid sequence change.

Some years ago Chesebro et al. generated transgenic mice lacking the PrP GPI signal sequence on a PrP null background (tgGPI^−^ mice) [60]. In mice hemizygous for the transgenes, PrP devoid of the GPI anchor (GPI^−^-PrP) was expressed at about one quarter the level of PrP in wild-type controls, was largely unglycosylated and absent from the plasma membrane. Nonetheless, these mice, inoculated with RML or 22L prions accumulated high levels of infectivity and PK-resistant PrP in the form of extracellular amyloid plaques in their brains, but exhibited no striking clinical signs up to 600 and 400 days post infection (dpi), respectively. GPI^−^-PrP^C^ to GPI^−^-PrP^Sc^ conversion is believed to occur extracellularly [11], [60]–[62]. Mice homozygous for the transgenes expressing GPI^−^-PrP at a twofold higher level than their hemizygous counterparts developed neurological signs from 300 to 480 dpi [63]. Cultured cells expressing GPI^−^-PrP secreted the bulk of the anchorless PrP into the medium, none was found at the cell surface but some was associated with membrane fractions [64]. Cells expressing epitope-tagged GPI^−^-PrP exposed to 22L prions did not become chronically infected, however during the acute infection phase, tagged PrP^res^ was transiently formed [61], [65].

To determine whether prions formed from PrP^C^ that had the same sequence but different post-translational modifications would retain their strain characteristics, we inoculated tgGPI^−^ mice with various prion strains or isolates and determined the cell tropism of the resulting isolates by the Cell Panel Assay (CPA) [33]. We found that the CPA properties of 22L prions remained unchanged, whereas those of RML, 139A, 79A and ME7 prions became distinctly different when propagated in tgGPI^−^ mice. RML, 139A and 79A prions are derived from drowsy goat scrapie and are related [66], [67] while ME7 and 22L are distinct and unrelated strains derived from sheep scrapie [68]. Because the changes in properties could have been due to the absence of the GPI anchor and/or to diminished glycosylation, RML and ME7 prions propagated in GPI^−^ mice were transferred to wild-type mice and their properties were again assessed. As judged by the CPA, ME7 prions recovered their original properties after one passage, showing that their adaptation to the GPI^−^ environment was readily reversible. RML-derived prions, however, did not recover their original cell tropism, even after three cycles of propagation in wild-type mice, and therefore constituted a novel, stable strain, which we designated “SFL” (“Scripps Florida Laboratory”). Because both RML and SFL prions were stably propagated in wild-type mice, we propose that the two conformations are separated by a high activation energy barrier which is abrogated in tgGPI^−^ mice, either because of the different structure or reduced expression level of the hypoglycosylated GPI-less PrP^C^, or because of a different replication mode in the extracellular space.

## Results

### Inoculation of tgGPI^−^ mice with various prion strains

TgGPI^−^ mice express a PrP construct in which a nonsense codon replaces that for serine233, the residue to which the GPI moiety is added in wild-type PrP; thus, the polypeptide chains are identical in the transgenic and wild-type mice [60].

TgGPI^−^ mice [60] (GPI^−^ for short) and C57BL/6 wild-type mice (C57 for short) were inoculated with the mouse-adapted scrapie prion strains 22L, RML, 79A, 139A and ME7 to give GPI^−^[22L], GPI^−^[RML], GPI^−^[79A], GPI^−^[139A] and GPI^−^[ME7] prions (Table S1). GPI^−^[ME7] and GPI^−^[RML] brain homogenate was injected into GPI^−^ mice for a second round of transmission, to give GPI^−^/GPI^−^[ME7] and GPI^−^/GPI^−^[RML] mice. Disease signs in GPI^−^ mice occurred later than in C57 mice; for example, RML caused terminal disease at >300 days after inoculation (dpi) in GPI^−^, and at about 142 dpi in C57 mice, similar to the values reported previously [63]. GPI^−^[RML] elicited disease in GPI^−^ mice after 263±47 dpi, suggesting adaptation of the RML prions to the novel environment.

### Comparative quantitation of PrP^res^ by Western blotting and sandwich ELISA

**Figure 1:**
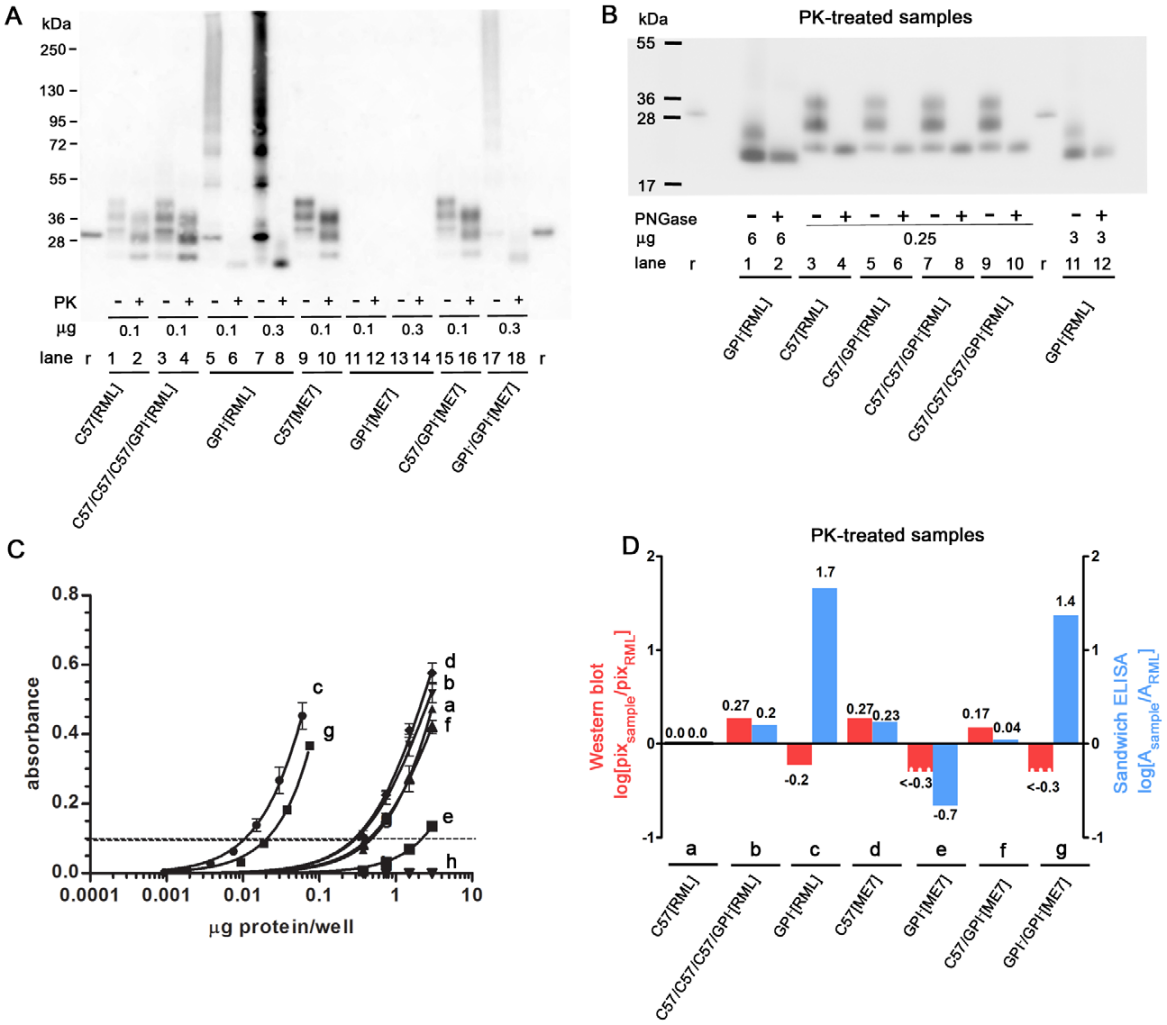
PrP^res^ in RML- and ME7-infected wild-type and tgGPI^-^ mouse brains, quantified by Western blot and sandwich ELISA. (A) Western blot of RML^−^ and ME7-infected brain homogenates before and after PK treatment. “µg”, total protein loaded. ‘r’, 2.5 ng recombinant murine PrP (recPrP). GPI^−^[RML] (lanes 5, 7) and GPI^−^/GPI^−^[ME7] (lane 17) brain homogenates gave rise to ladders reflecting multimers with molecular weights extending to >250 kDa, which were reduced to monomers by PK digestion, while GPI^−^[ME7] homogenate gave no detectable signals. (B) Western blot of PK-digested brain homogenates treated or not with PNGase. Lanes 3, 5, 7, 9 show, from top to bottom, diglycosylated, monoglycosylated and unglycosylated, truncated PrP; PNGase-treated samples (lanes 4, 6, 8, 10) show a single band corresponding to unglycosylated, truncated PrP. Samples from tgGPI^−^ mice (lanes 1, 11) show two bands, corresponding to truncated, monoglycosylated (top) and unglycosylated PrP, and, after PNGase treatment, a single band corresponding to truncated, deglycosylated PrP (lanes 2, 12). Because anchorless PrP is retained inefficiently by PVDF membranes [69], 24 times more total protein was loaded for GPI^−^ than for C57 samples, to give about the same signal strength. (**C**) Sandwich ELISA of PK-treated samples. Absorbance of quadruplicate samples is plotted against log[input protein]. Samples a to g are identified in panel D; h, uninfected C57 brain homogenate. The abundance of a sample relative to that of RML can be read off by comparing the amounts of protein required to give the same absorbance. For example, an absorbance of 0.1 is given by 0.01 µg GPI^−^[RML] and 0.5 µg C57[RML] brain homogenate (total protein prior to PK treatment), therefore the abundance of GPI^−^[RML] is about 50 times higher than that of C57[RML] PrP^res^. In Figure S2 absorbance of the same samples is plotted against input protein on a linear scale, to show that the response is almost linear up to a protein input of 1.5 µg/well. (D) The PrP^res^ signals (“pix”) from the western blots of Figure 1A were quantified relative to C57[RML] and the log of the ratio was plotted (red bars). For the sandwich ELISA, the plot shows the log of the absorbance (A) relative to that of C57[RML] (blue bars).

We first analyzed the prion-infected brain samples for their content of PrP^res^. Western blot analysis of C57[RML] and C57[ME7] brain homogenates showed three PrP-specific bands, attributed to di-, mono- and unglycosylated species, whose mobility increased after truncation by PK digestion (Figure 1A, lanes 1,2 and 9,10 respectively). The same amount or threefold higher of GPI^−^[ME7] sample gave no detectable PrP signal (lanes 11–14), while GPI^−^/GPI^−^[ME7] (lane 17) and GPI^−^[RML] (lane 5) samples gave rise to a ladder of bands with mobilities corresponding to about 28 to >250 kDa. Treatment with PK converted the ladders to 2 bands (lanes 6, 8, 18), the major one corresponding to unglycosylated and the minor one to monoglycosylated GPI^−^PrP, as shown by the fact that PNGase digestion abrogates the slower-moving band (Figure 1B, lanes 2 and 12). The aggregated forms of GPI^−^-PrP are likely derived from the abundant amyloid plaques described earlier [60]. Although GPI^−^[ME7] brain homogenate from mice culled at 300 dpi showed barely a trace, if any at all, of PrP^res^ on western blots (Figure 1A, lanes 12, 14) it nonetheless caused clinical disease by about 170 and 450 dpi when inoculated into wild-type and GPI^−^ mice, respectively (Table S1, #20, 21), raising the suspicion that quantitation by western blot was erroneous. Nishina and Supattapone have reported that PrP^C^ deprived of a GPI anchor by PIPLC-treatment was retained by the PVDF membranes used for western blotting at less than about 5% the efficiency of its GPI-linked counterpart [69]. We therefore compared the levels of PrP^res^ in PK-treated samples from GPI^−^ and C57 mice by western blotting and by sandwich ELISA, in which PK-treated, denatured samples were bound to wells coated with anti-PrP antibody D18 and visualized with biotinylated antibody D13. Astonishingly, sandwich ELISA revealed levels of PrP^res^ in GPI^−^[RML] and GPI^−^/GPI^−^[ME7] brain 50- and 25-fold higher, respectively, than those in C57[RML] brain (Figure 1C) rather than 30% and >50% lower, as indicated by western blotting (Figure 1A). Figure 1D shows that quantitation of PrP^res^ by western blotting and sandwich ELISA are consistent for wild-type brain but highly discordant for GPI^−^ brain. Moreover, sandwich ELISA showed that the GPI^−^[ME7] samples, which were negative by western blotting, in fact contained PrP^res^ at about 12% the level in C57[ME7] brains. Brains from GPI^−^ mice infected with 22L, 79A and 139A were not examined by western blotting or sandwich ELISA.

In summary, using the sandwich assay rather than western blotting for the quantitation of PrP^res^, it became evident that RML and ME7 prions propagating in GPI^−^ mice gave rise to unusually high levels of PrP^res^, likely due to greater stability of extracellular as compared to intracellular PrP^res^.

### Characterization of GPI^−^[RML] and C57[RML] prions by the CPA

Homogenates of prion-infected brains from GPI^−^ and C57 mice were analyzed by the CPA. In this assay, the cell lines CAD, PK1, LD9 and R33_2H11_ are infected with serial dilutions of a prion preparation and the proportion of infected cells is plotted against the logarithm of the dilution. The Response Index (RI) is defined as the dilution of the prion preparation at which an arbitrary proportion of cells (in this paper usually 3%) becomes PrP^res^ positive. The ratio of RI's for a prion preparation on a set of cell lines is a characteristic strain property [2]. Additional valuable strain discrimination is provided by the glycosylation inhibitors swainsonine (swa), kifunensine (kifu) and castanospermine (csp), which inhibit chronic infection of PK1 cells by various strains to different extents [70]. Swa [70]–[72] and kifu [71] are potent and selective inhibitors of class II and class I α-mannosidases, respectively, and lead to replacement of complex N-glycans by high-mannose glycans. Csp [72], by inhibiting glucosidases, causes replacement of complex glycans mainly by glucose-containing, high-mannose oligosaccharides.

**Figure 2:**
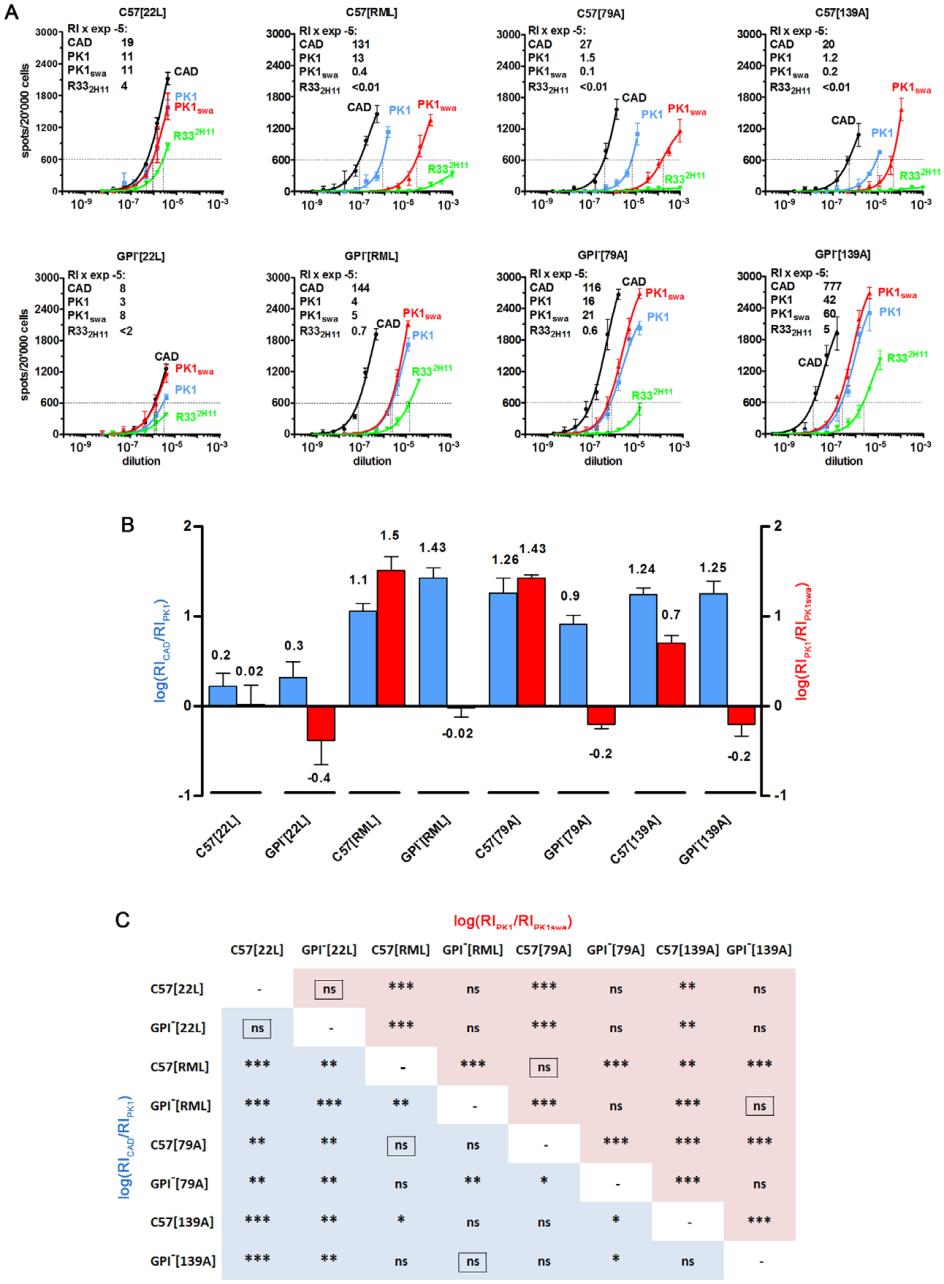
The cell tropism of various prion strains changes after propagation in tgGPI^−^ mice. Homogenates of GPI^−^ or C57 brains infected with the strains indicated were subjected to the CPA. (**A**) The patterns elicited by 22L from both sources were very similar, however RML, 79A and 139A prions from wild-type brain were swa sensitive on PK1 cells and R33^2H11^ incompetent, while those from tgGPI^−^ brain were swa resistant and R33^2H1^ competent. The RI_600_ (Response Index for 600 spots) on CAD, PK1, PK1_+swa_ and R33^2H11^ cells is given within the graphs (left upper corner) and the logarithm ± SD of the ratios RI_CAD_/RI_PK1_ (blue) and RI_PK1_/RI_PK1+swa_ (red) is plotted in the bar graph (B). The matrix (C) gives the p values for the pairwise comparison of two strains on the basis of their log[RI_CAD_/RI_PK1_] (blue) and log[RI_PK1_/RI_PK1+swa_] (red) values. The framed “ns” indicates p values>0.1 for *both* log[ratios]. For example, C57[RML] and GPI^−^[RML] prions are significantly different (p = 0.0097 for log[RI_CAD_/RI_PK1_] and p = 0.0001 for log[RI_PK1_/RI_PK1+swa_]), as are C57[79A] and GPI^−^[79A] prions, whereas C57[22L] and GPI^−^[22L] prions do not show a significant difference (framed “ns”; p>0.1) for both logRI ratios. By the same token C57[79A] and C57[RML], and GPI^−^[139A] and GPI^−^[RML] prions are not distinguishable, while C57[139A] and C57[RML] prions differ.

As shown by the CPA in Figure 2A, RML prions from C57 brain were swa sensitive on PK1 cells and R33^2H11^ incompetent, i.e. unable to infect R33^2H11^ cells efficiently, while RML-derived prions from GPI^−^ brain were swa resistant and R33^2H11^ competent; moreover, the RI_CAD_/RI_PK1_ ratio was lower for the C57-derived than for the GPI^−^-derived samples. The bar diagram (Figure 2B) shows log[RI_CAD_/RI_PK1_] (blue) and log[RI_PK1_/RI_PK1+swa_] (red) values plotted for 22L, RML, 79A, and 139A, propagated in C57 or GPI^−^ brain. The quantified data clearly show that C57[RML] and GPI^−^[RML] give vastly different patterns, as do their 79A and 139A counterparts. The statistical significance of the “log[ratio]” differences between two strains is given in the matrix of Figure 2C. For example, the difference between C57[RML] and GPI^−^[RML] is highly significant for both ratios, whereas there is no significant difference between C57[22L] and GPI^−^[22L] by the criteria used here. Interestingly, the differences between C57[RML] and C57[139A] are significant for both ratios, but not those between C57[RML] and C57[79A], contradicting the common assumption that RML and 139A are the same strains [67] and confirming the cognate conclusions of Browning et al. [73] and Oelschlegel et al. (personal communication).

From the RI values shown in the Figures and the PrP^res^ content relative to RML (Figure 1D) the relative specific infectivities (RI/PrP^res^) of RML and ME7 prions (second passage) derived from GPI^−^ mice were calculated and found to be 6- and 25-fold lower, respectively, than those from C57 mice (Table 1), suggesting that PrP^res^ accumulating extracellularly was either inherently less infectious or lost infectivity over time.

**Table 1:** Relative specific infectivity of various prion species.

				Relative specific infectivity	
	Prion species	Relative RI ± SD*	Relative PrPres ± SD+	RI/PrP^res^ ± SD#	Comparison to wild-type prions by the t test (p values)
A	C57[RML]	1.0±0.4	1.0±0.06	1.0±0.4	-
	GPI^−^[RML]	7.26±3.03	45.7±6.83	0.16±0.07	0.006
	C57/C57/C57/GPI^−^[RML]	1.05±0.46	1.62±0.32	0.65±0.31	ns
B	C57[ME7]	1.0±0.33	1.0±0.06	1.0±0.34	-
	GPI^−^[ME7]	0.04±0.014	0.13±0.012	0.28±0.11	0.0069
	GPI^−^/GPI^−^[ME7]	0.51±0.23	13.8±0.92	0.04±0.02	0.0013
	C57/GPI^−^[ME7]	0.58±0.22	0.64±0.112	0.91±0.38	ns

ns, not significant. All determinations were in quadruplicate.

*: RI's were determined on PK1 cells (A) or LD9 cells (B) by the SSCA and expressed relative to the values found for C57[RML] (A) or C57[ME7] (B).

**+:** PrP^res^ was determined by Sandwich ELISA as shown in Figure 1C and expressed relative to the values found for C57[RML] (A) or C57[ME7] (B).

# Relative specific infectivity is the ratio RI/PrP^res^ relative to that of C57[RML] (A) or C57[ME7] (B).

The specific infectivity of GPI^−^[RML] is 6 times lower than that of C57[RML] prions, and that of GPI^−^/GPI^−^[ME7] is 25 times lower than that of C57[ME7] prions, underlining the changed properties of prions adapted to GPI^−^ brain. After being returned to and propagated in wild-type brain, C57/C57/C57/GPI^−^[RML] prions (which show a novel, stable CPA response and constitute a novel strain, designated SFL) do not fully regain the specific infectivity of RML, however the difference is not statistically significant. ME7 prions fully regain the original specific infectivity on being returned from GPI^−^ to wild-type mice.

### Transfer of GPI^−^ [RML] prions to C57BL/6 mice

Because PrP^C^ and PrP^res^ in GPI^−^ mice lack the GPI anchor and are also largely unglycosylated (Figure 1B) [1], the question arose whether the differences in the CPA characteristics of GPI^−^-derived and wild-type prions were due to these structural features (which we considered unlikely because GPI-linked PrP^res^ arises immediately after infecting the cells for the CPA) or to a conformational change of the PrP^res^. We therefore inoculated brain homogenate from GPI^−^[RML] mice into C57BL/6 mice to generate C57/GPI^−^[RML] prions, whose PrP^res^ then carried a GPI anchor and was normally glycosylated (Figure 1B, lanes 5). The resulting brain homogenate was serially transmitted twice more to C57BL/6 mice, to yield C57/C57/GPI^−^[RML] (Figure 1B, lanes 7, 8) and C57/C57/C57/GPI^−^[RML] prions (Figure 1B, lanes 9, 10; Figure 1A, lanes 3, 4).

**Figure 3:**
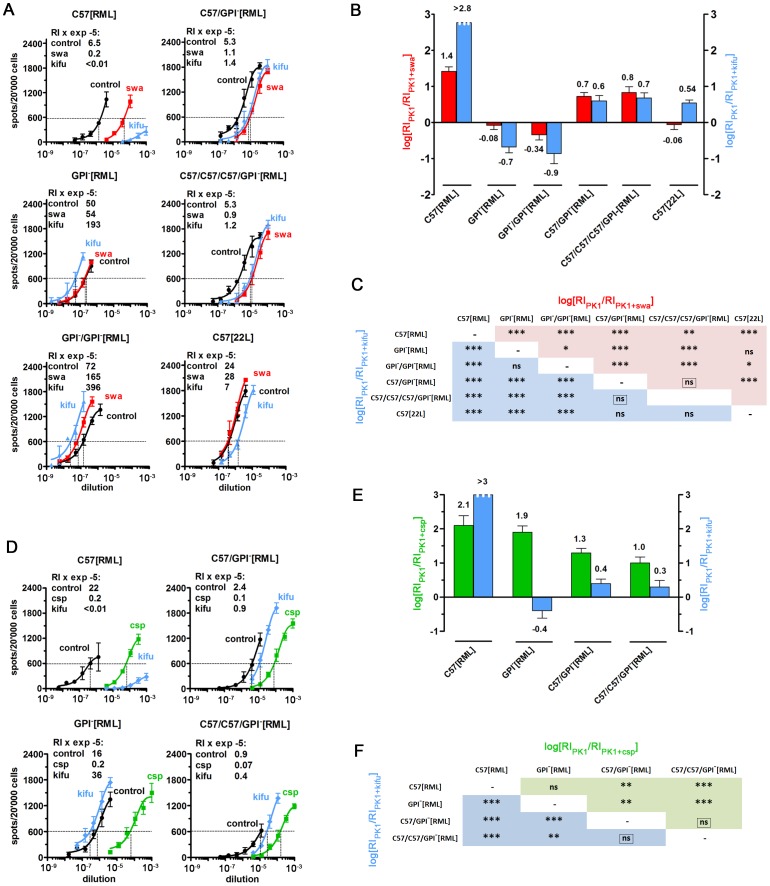
RML prions propagated in tgGPI^−^ mice and returned to wild-type mice emerge as a novel, stable strain. Analysis of authentic RML prions propagated in C57BL/6 brain (C57[RML]), and of RML prions first propagated in GPI^−^ brain (GPI^−^[RML]) and then once (C57/GPI^−^[RML]), twice (C57/C57/GPI^−^[RML]) or three times (C57/C57/C57/GPI^−^[RML]) in C57BL/6 brain. (A) shows the SSCA performed on PK1 cells in the presence or absence of kifunensine (kifu; 5 µg/ml) or swainsonine (swa; 2 µg/ml) for the samples indicated; C57[22L] was added as control. RI's were determined at 600 spots (RI_600_). Kifu strongly inhibited the propagation of C57[RML], but not of C57/GPI^−^[RML] and C57/C57/C57/GPI^−^[RML] prions on PK1 cells. (B) The bar graph shows the log[RI_PK1_/RI_PK1+kifu_] and log[RI_PK1_/RI_PK1+swa_] values for the samples listed in (A). The pairwise comparison in panel (C) shows that C57/GPI^−^[RML] and C57/C57/C57/GPI^−^[RML] prions do not differ significantly, but are vastly different from C57[RML] and GPI^−^[RML] prions. The framed “ns” indicates high p values (>0.1) for *both* log[ratios], indicating no significant difference between the samples. (D) Effect of castanospermine (csp; 50 µg/ml) or kifu (5 µg/ml) on propagation of the samples indicated on PK1 cells. As in (A), kifu strongly inhibits the propagation of C57[RML], but not C57/GPI^−^[RML] and C57/C57/GPI^−^[RML] prions. The same is true for csp, however to a lesser extent. (E) The bar graph depicts the log[RI_PK1_/RI_PK1+kifu_] (blue) and log[RI_PK1_/RI_PK1+csp_] (green) values. The RI_PK1_/RI_PK1+kifu_ ratio was >500-fold lower for C57/GPI^−^[RML] and C57/C57/GPI^−^[RML] than for C57[RML] prions, again underscoring the difference between RML prions and the novel strain. The matrix (F) also shows that C57/GPI^−^[RML] and C57/C57/GPI^−^[RML] prions do not differ from each other, but that both differ significantly from C57[RML] prions. In summary, the figure sustains the conclusion that a new strain, designated SFL, emerged after RML prions were passaged through GPI^−^ brain and returned C57 brain, and that they remained unchanged after two further passages.

C57BL/6 mice inoculated with prions from GPI^−^[RML] brain exhibited pronounced clinical signs of RML scrapie disease after 150±9 days, similar to those of mice inoculated with the original C57[RML] (Table S1, #4a,b; #1). However, as shown in Figure 3A, C57/GPI^−^[RML] prions neither retained the drug susceptibility pattern of GPI^−^[RML], nor did they regain the pattern characteristic for the original C57[RML] prions, even after two additional transfers through C57 mice. From the bar diagram (Figure 3B) it can be seen that the log[RI_PK1_/RI_PK1+kifu_] (blue) and log[RI_PK1_/RI_PK1+swa_] (red) values for C57/GPI^−^[RML] and C57/C57/C57/GPI^−^[RML] prions, 0.6, 0.7, and 0.7, 0.8, respectively, were indistinguishable, but far lower than those for C57[RML] prions, >2.8 and 1.4, respectively. The statistical evaluation shown in the matrix of Figure 3C confirms the significance of these conclusions.

In a further experiment (Figure 3D), the susceptibility of prion replication in PK1 cells to inhibition by csp and, again, kifu was tested. The bar diagram (Figure 3E, blue bars) shows that kifu, as before, inhibited infection of PK1 cells by RML by more than 3 logs but had little effect on infection by C57/GPI^−^[RML] or C57/C57/GPI^−[^RML] prions, and Figure 3F documents the statistical significance of the conclusions. In addition, we cloned RML prions in PK1 cells, propagated them in mouse brain and determined that none of twelve RML subclones exhibited kifu resistance, excluding the possibility that SFL was a significant component of RML (Supporting Information, Figure S1). Csp inhibited RML infection of PK1 cells by about 2 logs, but that of GPI^−^-derived prions passaged once or twice in C57 mice by only about 1 log (Figure 3E, green bars). Thus, as judged by the CPA and by inhibitor susceptibility, RML prions passaged through GPI^−^ brain acquired novel characteristics and these were retained even after three serial passages through C57BL/6 brain.

The conformational stability assay [26] showed no difference between the PrP^res^ associated with wild-type RML, C57/GPI^−^[RML] and C57/C57/GPI^−^[RML], however GPI^−^[RML] PrP^res^ seemed slightly more stable; whether this reflects a conformational difference or a difference due to the absence of GPI anchor and paucity of N-glycans remains unknown (Figure S2).

### Characterization of GPI^−^[ME7] and C57[ME7] prions by the CPA

**Figure 4:**
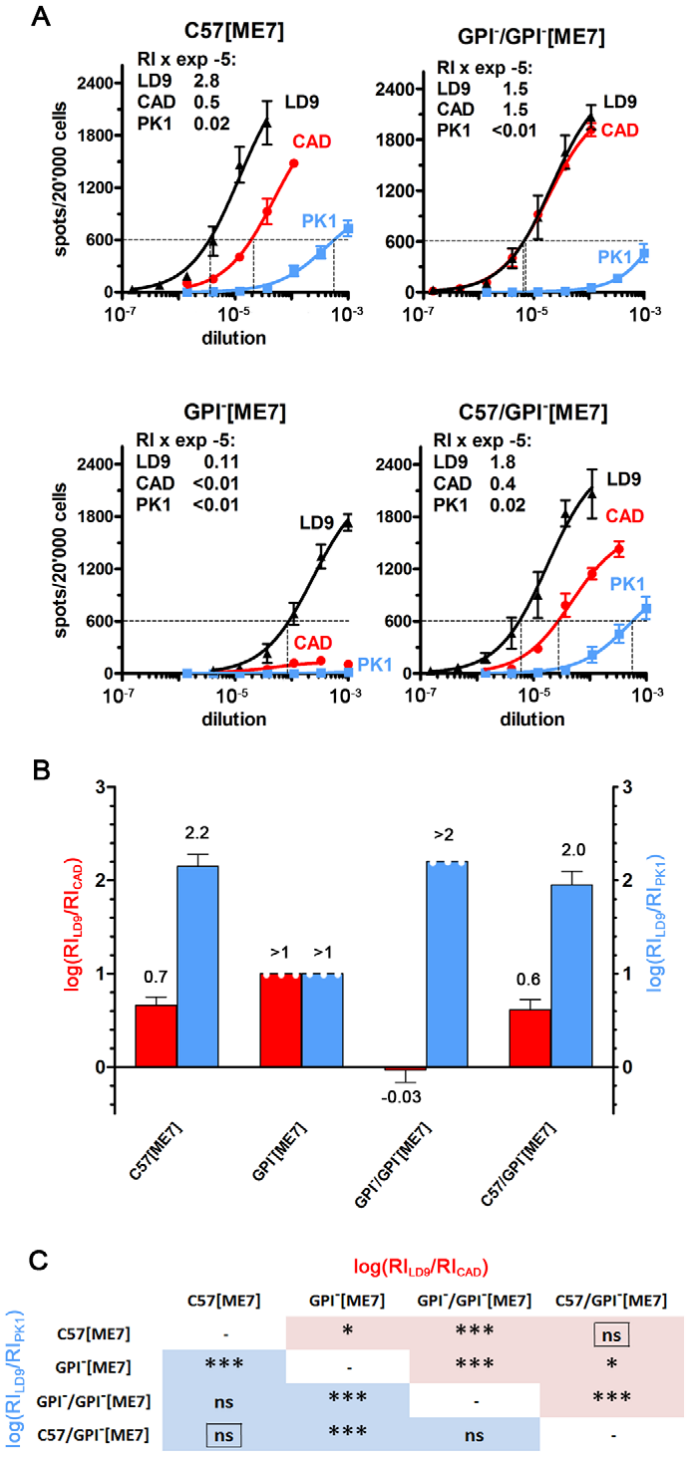
ME7 prions propagated in tgGPI^−^ mice for one or two passages acquire novel characteristics. (A) Serially diluted brain homogenates were analyzed on LD9, CAD and PK1 cells by the CPA. After the first passage of ME7 prions in GPI^−^ mice (GPI^−^[ME7]), the RI on CAD cells dropped 20-fold, most likely reflecting low titers, but increased about 30-fold after the second passage (GPI^−^/GPI^−^[ME7]), indicating adaptation to the GPI^−^ environment. Returning prions from the first passage in GPI^−^ brain to wild-type brain (C57/GPI^−^[ME7]) restored the original CPA pattern. (B) Log[RI_LD9_/RI_CAD_] (red) and log[RI_LD9_/RI_PK1_] (blue) are plotted as a bar graph. (C) The matrix shows that C57[ME7] and C57/GPI^−^[ME7] prions do not differ, while both differ in at least one log[ratio] from GPI^−^[ME7] and GPI^−^/GPI^−^[ME7] prions.

Typical scrapie signs were not observed in ME7-inoculated GPI^−^ mice by 300–305 days, at which time mice showing mild clinical signs were euthanized and homogenates of their brains were subjected to the CPA. The level of infectivity of GPI^−^[ME7] brain homogenates as measured on CAD and PK1 cells was very low (RI<10^3^), however LD9 cells showed a higher response (RI = 1.1×10^4^), albeit at a level about 25 times lower than that found for C57[ME7] brains 2.8×10^5^ (Figure 4). The GPI^−^[ME7] brain homogenates were infectious to wild-type mice, leading to disease at 170±0 dpi (Table S1, #20), as compared to 138±4 days for “normal” C57[ME7] (Table S1, #18b), and yielded prions indistinguishable from the original ME7, as judged by the CPA. A second transmission of GPI^−^[ME7] prions to GPI^−^ mice resulted in disease at 447±64 dpi (Table S1, #21) and, interestingly, to high levels of PrP^res^, as determined by sandwich ELISA (Figure 1C, 1D), and infectivity, as measured on CAD cells (RI = 1.5×10^5^; Figure 4A). GPI^−^/GPI^−^[ME7] prions differed from C57[ME7] prions by their log[RI_LD9_/RI_CAD_] value, -0.03 versus 0.7; whether or not they revert to the original ME7 after being returned to C57 is still under investigation (Table S1, 22.) (Figure 4B, C). In summary, repeated propagation of ME7 prions through GPI^−^ brain resulted in a distinct alteration of their properties, reflecting progressive adaptation to the modified environment. However, prions returned from GPI^−^[ME7] mice to C57 mice resulted in reversion to prions indistinguishable from the original C57[ME7] prions.

## Discussion

Prion populations are considered to be “quasi-species”, i.e. to consist of a major component and a multiplicity of variants present at low levels, of which one may be selected as the major component if the population is exposed to a different environment [39], [74], [75].

We have reported previously that when 22L prions were transferred from brain to PK1 or R33 cells, their properties, as measured by the CPA, changed gradually in the course of many doublings, suggesting that a 22L variant present at low levels in the brain-derived population was being selected in the cellular environment. Conversely, when the cell-adapted 22L variants were again propagated in brain, the resulting population gradually re-acquired the properties of the original brain-derived 22L. When 22L prions were cloned by endpoint dilution into PK1 cells they were initially swa sensitive and incapable of developing swa resistance (“swa incompetent”), but as the populations were further propagated they became swa competent while remaining swa sensitive, suggesting that during propagation swa-resistant prions arose at a low level by “mutation” and could be selected when the population was challenged with swa [74]–[76].

**Figure 5:**
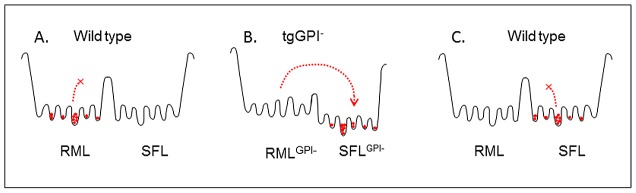
Conjectural free energy profile. (A) The RML quasi-species in wild-type brain are confined to a set of wells separated from those of the SFL quasi-species by a high activation energy barrier. (B) In tgGPI^−^ brain the transition to the “SFL^GPI-^” set of conformations is enabled by a lower activation energy barrier. (C) SFL^GPI-^ prions introduced into wild-type brain can now occupy, and are trapped in, the set of “SFL wells” which was not accessible to RML prions in (A).

In all these cases the changes were reversed when the prions were propagated in the original environment; assuming that properties of prions are encoded by the precise conformation of the cognate PrP^Sc^, we concluded that the conformational states underlying adaptation to the cellular environment are separated by low activation energy barriers which allowed reversible conformational switches leading to “strain variants” or “sub-strains” [75]. In contrast to these earlier results with 22L prions, we now report that when RML prions from wild-type brain were propagated in brain producing anchorless PrP, a GPI^−-^PrP^Sc^ conformer adapted to that environment evolved, and when this was transferred to wild-type mice, a novel conformation of wild-type PrP^Sc^, which we designated SFL, distinct from that of the original RML, was stably maintained. Because SFL does not revert to RML, it must be either better adapted to propagation in wild-type brain, prevented from reverting by a high activation energy barrier or both (Figure 5). Of note, methods commonly used to distinguish strains, such as incubation period, western blotting or conformational stability assays could not differentiate between RML and SFL, whereas this was readily achieved with the Extended Cell Panel Assay (ECPA) [77]. ME7 also acquired distinct properties when propagated in tgGPI^−^ brain, however it reverted to what appears to be its original form when passaged through wild-type brain. Strain switching has been observed previously in transfer between animal species, when 139A prions were transferred from mouse to hamster and back to mouse [78]; in that case, a different amino acid sequence in the intermediate host may have led to the adoption of a more favorable conformation, which was preserved when the prions were returned to the original host. Mutated PrP^Sc^ could be formed if accretion of GPI^−^-PrP^C^ to wild-type seed entailed adoption of a conformation slightly different from that of the seed, or if wild-type PrP^Sc^ contained a variety of mutant seeds –possibly at a low level– to one or some of which the resident GPI^−^PrP^C^ preferentially accreted, adopting its conformation [76].

Considering the large variety of “classical” murine prion strains [79], the plethora of strains generated by Prusiner and his colleagues [80], [81] and the many variants we have observed, all encoded by a single murine PrP sequence, the number of stable conformational states must be vast. The structural elucidation of PrP^Sc^ and its variants continues to be the Holy Grail of the prion field.

## Materials and Methods

### Ethics statement

When working with mice all efforts were made to minimize suffering. This study was carried out in strict accordance with the recommendations in the Guide for the Care and Use of Laboratory Animals of the National Institute of Health. The protocol was approved by the Institutional Animal Care and Use Committee (IACUC). Scripps Florida has an Animal Welfare Assurance on file with the Office of Laboratory Animal Welfare (OLAW), National Institute of Health (assurance number #A4460-01). Scripps Florida's registration under USDA regulations is certificate 93-R0015. The Association for Assessment and Accreditation of Laboratory Animal Care International (AAALAC) awarded Scripps Florida full accreditation.

### Mouse inoculations

C57BL/6 mice were from Charles River Laboratories (Wilmington, MA). Breeding pairs of tgGPI^−^ mice [60] were obtained from the Oldstone laboratory. Mice were anesthetized by isoflurane and 30-µl samples of 1% brain homogenates were inoculated in the prefrontal cortex. When pronounced clinical signs became evident, or earlier, the mice were asphyxiated with CO_2_ and subjected to cervical dislocation.

### Prion preparations

The 22L strain, cloned by two successive end-point dilutions, as well as strains 79A and 139A were obtained from the TSE Resource Centre, Compton, Newbury, UK (I. McConnell, R.M. Barron). The RML strain, from the MRC Prion Unit, University College, London, was propagated initially in CD1 mice and subsequently in C57BL/6 mice.

Brains were harvested from mice exhibiting neurological signs of prion diseases in C57BL/6. In some cases where inoculated, anchorless mice showed only mild clinical signs, brains were taken at 300 days post inoculation. For stock homogenates, frozen brains were pooled and homogenized for 10 sec in PBS (9 ml per g) using a hand-held Ultramax T18 basic homogenizer (IKA Works Inc., Bloomington, NC) at 20000–25000 rpm. Single frozen brains were homogenized in PBS using a ribolyser (FastPrep FP120, Bio 101, Thermo Electron Corp.,Thermo Fischer Scientific, MA) with ZrO 0.8–1 mm beads (cat. No. 7305-000010, Glen Mills Inc. Clifton, NJ), at maximum speed 6.5 for 15 seconds in Fast Prep tubes (MP Biomedicals).

### Cell culture

The cell lines PK1 and R33_2H11_ are derived from N2a neuroblastoma cells, CAD5 (CAD) from Cath.a-differentiated cells, and LD9 from L929 fibroblasts [33], [82]. The lines were maintained in OBGS (Opti-MEM [Invitrogen], 4.5% Bovine Growth Serum [Hyclone, Logan, UT], 90 units penicillin/ml, 90 µg streptomycin/ml [Invitrogen]). Cells were split 1∶10, or 1∶8 if sparse, twice a week. After 9 serial passages cells were replaced with freshly thawed samples.

### The Standard Scrapie Cells Assay (SSCA)

The SSCA was performed as detailed earlier [82]. In summary, serial 1∶3 dilutions of the sample (300 µl) were placed in quadruplicate wells in 96-well plates with 5000 susceptible cells per well. Four days after infection the confluent monolayer was suspended and split 1∶7 in OBGS for total 3 splits, allowing cells 3–4 days to form a confluent layer between each split. After the third split, cells were grown to confluence again, 20000 cells were added to the wells of pre-activated Multiscreen IP96-well 0.45-µm Immobilon P membrane plates (Millipore, Danvers, MA) and vacuumed onto the membrane. After baking at 50°C for 1 h, samples were treated with 1 µg proteinase K (PK, Roche)/ml in lysis buffer, followed by PMSF and denaturation with guanidinium thiocyanate. In more recent assays the PMSF step was omitted. After washing with PBS, wells were blocked with 0.5% milk in 1× TBS and incubated with humanized anti-PrP antibody D18 [83] followed by mouse anti-human IgGγ AP-conjugated antibody in 0.5% milk-TBST. Signals were visualized with AP Conjugate Substrate Kit (BioRad) and PrP^res^-positive cells (“spots”) were counted using the Bioreader 5000-Eb (BioSys). To control for residual inoculum, the prion replication inhibitor pentosan polysulfate (PPS) was added during the 4-day infection at 10 µg/ml to the cells infected with the highest concentration of prion sample.

The number of PrP^res^-positive cells is plotted as a function of the logarithm of the dilution. The “Response Index” (RI) of a sample is the reciprocal of the concentration that gives rise to a designated proportion of PrP^Sc^-positive cells under standard assay conditions (in our experiments, RI_600_, 600 positive cells per 20000 cells, or 3%).

### Cell Panel Assay

The Cell Panel Assay (CPA) allows the characterization of strains by virtue of their cell tropism and their susceptibility to inhibitors, as determined by the SSCA performed on CAD5, PK1, LD9 and R33_2H11_ cells. A strain or a substrain is characterized by the ratio of RIs on different cell lines and by the susceptibility to inhibition by drugs. When required, glycosylation inhibitors swainsonine (swa, Logan Natural Products; 2 µg/ml), kifunensine (kifu, Toronto Research Chemicals Inc.; 5 µg/ml) and castanospermine (csp, Toronto Research Chemicals Inc.; 50 µg/ml) were present during infection and up to the second split, and were diluted thereafter with each split.

### End-point dilution cloning of RML in cell culture

PK1 cells were seeded at 100 cells/well in 96-well plates and inoculated with highly diluted RML-infected brain homogenates (final concentration: 10^−9^; 5×10^−10^). The cells were repeatedly grown to confluence and subjected to three 1∶3 splits, followed by eight 1∶10 splits; after a total of about 50 doublings 20000 cells from each well were subjected to the PK-Elispot Assay and samples containing PrP^res^-positive cells (spot numbers>[background+5 SDs]) were scored as positive. The proportion of positive wells was 27/168 (16%) for an inoculum dilution of 10^−9^ and 27/252 (11%) for an inoculum dilution of 5×10^−10^; the probability P_N>1_ = 1 - e^−m^ (1+m) that under the conditions chosen a well was infected by more than one prion was 10^−2^–10^−3^ or less, where m is the average number of prions/well. Nine prion clones were expanded, conditioned media was harvested, concentrated and inoculated into C57BL/6 mice. The brain homogenates from terminally ill mice were characterized by the SSCA on PK1 cells in the presence or absence of 5 µg kifu/ml for all 9 clones from the 1^st^ round of cloning. In addition, prions from several RML-infected PK1 (PK1[RML]) clones were subjected to another round of end-point dilution cloning in cells. Eight clones of 2^nd^- round RML-infected PK1 (PK1{PK1[RML]}) cells were expanded, conditioned media was harvested, concentrated and inoculated into C57BL/6 mice. The brain homogenates from three of the eight terminally-ill mice were characterized by the SSCA on PK1 cells in the presence or absence of 5 µg kifu/ml.

### Western blot analysis

Samples were denatured by boiling in XT-MES sample buffer (BioRad), fractionated by SDS-PAGE on 4–12% Criterion gel (BioRad) for 1.5 h at 120 V and transferred to PVDF Immobilon membranes (Millipore) by wet transfer (Criterion, BioRad). Membranes were blocked in 5% non-fat dry milk/PBST and exposed to 0.5 µg D18 anti-PrP antibody/ml in 5% non-fat dry milk/PBST followed by mouse anti-human IgG HRP-conjugated secondary antibody (48 ng/ml, Southern Biotech) in 5% non-fat dry milk/PBST. Chemiluminescence was induced by ECL-Plus (Pierce) and recorded by CCD imaging (BioSpectrum AC Imaging System; UVP). PageRuler Plus Prestained Protein Ladder (Fermentas) was run as molecular weight marker.

### Conformational Stability Assay

The method is essentially that of Peretz et al. [23]. Prion-infected brain homogenates (30 µg total protein) were adjusted to between 0.5 M and 5 M guanidinium chloride (GndCl) in 10 mM Tris-HCl (pH 8.0) (final volume 60 µl) and incubated for 15 min at 25°C, shaking at 700 rpm in an Eppendorf thermomixer. The GndCl concentration in each sample was then adjusted to 0.2 M with 10 mM Tris-HCl (pH 8.0) and the volumes were equalized to 985 µl with 0.2 M GndCl in 10 mM Tris-HCl (pH 8.0). Samples were adjusted to 0.5% Triton X-100 and digested with 0.6 µg/ml PK (PK : protein = 1∶50 by weight) with shaking at 1000 rpm for 1 h at 37°C in an Eppendorf thermomixer; digestion was terminated by addition of PMSF to 2 mM. After addition of 5 µg BSA, proteins were precipitated with TCA (10% final concentration), chilled on ice for 30 min, and centrifuged 15 min at 16000× g and 4°C. Pellets were resuspended in 0.5 ml cold acetone, re-centrifuged and resuspended in 50 µl PBS-0.5% Triton X-100. Samples were heated 10 min at 100°C in MES sample loading buffer (Bio-RAD) and analyzed by western blotting. Chemiluminescence was induced by ECL-Plus (Pierce), recorded and quantified by CCD imaging (BioSpectrum AC Imaging System; UVP). The highest value of each curve was set to 100% and the Gnd_1/2_ value, i.e. the GndCl concentration at which 50% of the PrP^res^ was digested by PK under standard conditions, was determined.

### Sandwich ELISA

The procedure is essentially as described earlier [17]. Ninety-six-well plates (F16 Maxisorp Nunc Immune Module, Nunc) were rocked overnight with 15 µg/ml D18 antibody/well at 4°C, washed with PBST 3 times, blocked with 5% milk in PBST at 37°C for 1 h, washed again in PBST 3 times and stored with 200 µl PBS per well at 4°C. Brain homogenates were diluted to 3 mg total protein/ml in 0.5% Triton X-100. Samples with high PrP^res^ content were diluted appropriately with PrP^o/o^ brain homogenate to give a final protein concentration of 3 mg/ml. Samples were shaken with 12.5 µg proteinase K (PK)/ml at 37°C for 1 h, and adjusted to 6 mM PMSF, giving a protein concentration of 2.8 mg/ml. A 7.2-µl aliquot of each sample was denatured with an equal volume 8 M GndCl (Research Products International Corp.) for 5 min at 80°C and diluted in Sandwich Assay Buffer (50 mM Tris-HCl, pH 8.0, 2% Triton X-100, 2% sodium lauroylsarcosine, 2% BSA) to a final volume of 1 ml. Quadruplicate 300-µl sample aliquots were added to the wells of blank 96-well tissue culture plate (BD Falcon) and serially diluted 1∶2 in Sandwich Assay Buffer. When used as a standard, recombinant PrP (200 ng/ml in 2.8 mg/ml PrP^o/o^ brain homogenate) was serially diluted into 2.8 mg/ml PrP^o/o^ brain homogenate. A 150-µl aliquot of each sample was transferred to the D18-coated wells and rocked 1 h at 37°C. Plates were washed 5 times with PBST (0.1% Tween/PBS) and to each well was added 100 µl biotinylated D13 antibody [83] at 1.3 µg/ml 1% milk-PBST. After rocking for 1 h at 37°C in 1% milk-PBST, plates were washed 5 times with PBST and to each well was added 100 µl 1∶7500 HRP-streptavidin (Amersham GE Healthcare) in 0.5% BSA-PBST for 30 min at 37°C. After 5 washes with PBST, 100 µl TMB Super-sensitive HRP microwell substrate (SUB2) was added, and after 2 min at room temperature the reaction was stopped with 100 µl TMB Microwell Stop solution (STOP1; Immunochemistry Technologies, MN). Plates were read with a BioTek 450 plate reader (BioTek Instruments, VT) and analyzed with Gen5 software.

## Supporting Information

Figure S1 **Determination of kifunensine-susceptibility of twelve independent clones of RML.** PK1 cells were infected with a high dilution of RML prions as described in the Methods section. Prions from RML-infected clones were inoculated in C57BL/6 mice and serial dilutions of homogenized brains were analyzed by the SSCA on PK1 cells in the presence or absence of 5 µg kifu/ml. All 12 clones were indistinguishable from the original RML in being strongly inhibited by kifu. RML and the clones were distinctly different from SFL, which was only weakly inhibited by kifu. The SSCA of only one representative of the 12 clones (#3) is shown.
(TIF)

Figure S2 **Sandwich ELISA of samples from** **Figure 1A** **.** Absorbance of quadruplicate samples is plotted against input protein on a linear plot, to show that absorbance is almost linear with input protein up to1.5 µg/well. NBH, uninfected C57 brain homogenate.
(TIF)

Figure S3 **Conformational stability of PrP^res^ from various strains.** The assay was described in the Methods section. The highest value for each curve was set to 100% and the concentration of GndCl at which 50% of the PrP^res^ was digested by PK is represented by the dotted lines. PrP^res^ from RML (lilac), C57/GPI^−^[RML] (green) and C57/C57/GPI^−^[RML] (blue) showed no significant differences in stabilities, which ranged from 1.7 to 1.9 M GndCl. GPI^−^[RML] (red) had a marginally higher stability (2.2 M GndCl).
(TIF)

Figure S4 **Kinetic Protein Misfolding Cyclic Amplification (PMCA)**. PMCA was performed to identify differences in rate constants of amplification of PrP^res^ from C57[RML] and C57/GPI^−^[RML] brains. PMCA substrate (uninfected C57 brain homogenate) was prepared as described [84], but without centrifugation. Prion “seeds”, RML and SFL (C57/GPI^−^[RML]) PrP^res^ were adjusted to the same levels, as determined by densitometric analysis on western blots. (A) PMCA reaction mixtures contained 441 µl substrate and either 9 µl of a 10^−2^ dilution of RML or an equivalent amount of SFL PrP^res^. PMCA was performed with 60-µl aliquots of the reaction mixtures dispensed in triplicate into 200-µl PCR tubes (Axygen) containing 37±3 mg of 1.0 mm Zirconia/Silica beads (Biospec products). Samples were subjected to cycles of 20 second sonication and 30 min incubation at 37°C for 0, 0.6, 0.9, 1.5, 2.2, 3.3 or 5 h, using a Misonix 3000 sonicator at power setting 8.5. To measure amplified PrP^res^, 20-µl aliquots were PK-digested and 10 µl were electrophoresed and analyzed as previously described [73]. (B) Western blots were quantified and the ratio rPrP^res^(time t)/rPrP^res^(time 0) was plotted on a log scale against time. The rate constants were calculated from the logarithmic phases of the kinetics (up to 3.3 h). (C) There were no statistically significant differences (t student) in the rate constants of RML and C57/GPI^−^[RML] PrP^res^ amplification.
(TIF)

Table S1 **Incubation periods and clinical signs for C57BL/6 and tgGPI^−^ mice inoculated with various inocula.**
(DOCX)

Text S1 **Incubation periods, clinical signs, biochemical and infectivity analyses of prion strains in tgGPI- and wild-type C57BL/6 mice.**
(DOCX)

## References

[1] Endo T, Groth D, Prusiner SB, Kobata A (1989). Diversity of oligosaccharide structures linked to asparagines of the scrapie prion protein.. Biochemistry.

[2] Rudd PM, Endo T, Colominas C, Groth D, Wheeler SF (1999). Glycosylation differences between the normal and pathogenic prion protein isoforms.. Proc Natl Acad Sci U S A.

[3] Rudd PM, Wormald MR, Wing DR, Prusiner SB, Dwek RA (2001). Prion glycoprotein: structure, dynamics, and roles for the sugars.. Biochemistry.

[4] Beringue V, Mallinson G, Kaisar M, Tayebi M, Sattar Z (2003). Regional heterogeneity of cellular prion protein isoforms in the mouse brain.. Brain.

[5] Somerville RA, Hamilton S, Fernie K (2005). Transmissible spongiform encephalopathy strain, PrP genotype and brain region all affect the degree of glycosylation of PrPSc.. J Gen Virol.

[6] Monnet C, Marthiens V, Enslen H, Frobert Y, Sobel A (2003). Heterogeneity and regulation of cellular prion protein glycoforms in neuronal cell lines.. Eur J Neurosci.

[7] Borchelt DR, Rogers M, Stahl N, Telling G, Prusiner SB (1993). Release of the cellular prion protein from cultured cells after loss of its glycoinositol phospholipid anchor.. Glycobiology.

[8] Hegde RS, Mastrianni JA, Scott MR, DeFea KA, Tremblay P (1998). A transmembrane form of the prion protein in neurodegenerative disease.. Science.

[9] Hegde RS, Tremblay P, Groth D, DeArmond SJ, Prusiner SB (1999). Transmissible and genetic prion diseases share a common pathway of neurodegeneration.. Nature.

[10] Goold R, Rabbanian S, Sutton L, Andre R, Arora P (2011). Rapid cell-surface prion protein conversion revealed using a novel cell system.. Nat Commun.

[11] Campana V, Sarnataro D, Zurzolo C (2005). The highways and byways of prion protein trafficking.. Trends Cell Biol.

[12] Veith NM, Plattner H, Stuermer CA, Schulz-Schaeffer WJ, Burkle A (2009). Immunolocalisation of PrPSc in scrapie-infected N2a mouse neuroblastoma cells by light and electron microscopy.. Eur J Cell Biol.

[13] Marijanovic Z, Caputo A, Campana V, Zurzolo C (2009). Identification of an intracellular site of prion conversion.. PLoS Pathog.

[14] Stahl N, Baldwin MA, Burlingame AL, Prusiner SB (1990). Identification of glycoinositol phospholipid linked and truncated forms of the scrapie prion protein.. Biochemistry.

[15] Safar JG, Wille H, Itri V, Groth D, Serban H (1998). Eight prion strains have PrP(Sc) molecules with different conformations.. Nat Med.

[16] Pastrana MA, Sajnani G, Onisko B, Castilla J, Morales R (2006). Isolation and Characterization of a Proteinase K-Sensitive PrP(Sc) Fraction.. Biochemistry.

[17] Cronier S, Gros N, Tattum MH, Jackson GS, Clarke AR (2008). Detection and characterization of proteinase K-sensitive disease-related prion protein with thermolysin.. Biochem J.

[18] Bruce ME, Fraser H, McBride PA, Scott JR, Dickinson AG, Prusiner SB, Collinge J, Powell J, Anderton B (1992). The basis of strain variation in scrapie.. Prion Diseases of Humans and Animals.

[19] Baron GS, Hughson AG, Raymond GJ, Offerdahl DK, Barton KA (2011). Effect of glycans and the glycophosphatidylinositol anchor on strain dependent conformations of scrapie prion protein: improved purifications and infrared spectra.. Biochemistry.

[20] Bessen RA, Marsh RF (1992). Identification of two biologically distinct strains of transmissible mink encephalopathy in hamsters.. J Gen Virol.

[21] Collinge J, Sidle KC, Meads J, Ironside J, Hill AF (1996). Molecular analysis of prion strain variation and the aetiology of ‘new variant’ CJD [see comments].. Nature.

[22] Kuczius T, Groschup MH (1999). Differences in proteinase K resistance and neuronal deposition of abnormal prion proteins characterize bovine spongiform encephalopathy (BSE) and scrapie strains.. Mol Med.

[23] Peretz D, Scott MR, Groth D, Williamson RA, Burton DR (2001). Strain-specified relative conformational stability of the scrapie prion protein.. Protein Sci.

[24] Telling GC, Parchi P, DeArmond SJ, Cortelli P, Montagna P (1996). Evidence for the conformation of the pathologic isoform of the prion protein enciphering and propagating prion diversity.. Science.

[25] Bruce ME, McBride PA, Farquhar CF (1989). Precise targeting of the pathology of the sialoglycoprotein, PrP, and vacuolar degeneration in mouse scrapie.. Neurosci Lett.

[26] Fraser H, Dickinson AG (1973). Scrapie in mice. Agent-strain differences in the distribution and intensity of grey matter vacuolation.. J Comp Pathol.

[27] Hecker R, Taraboulos A, Scott M, Pan KM, Yang SL (1992). Replication of distinct scrapie prion isolates is region specific in brains of transgenic mice and hamsters.. Genes Dev.

[28] Rubenstein R, Deng H, Race RE, Ju W, Scalici CL (1992). Demonstration of scrapie strain diversity in infected PC12 cells.. J Gen Virol.

[29] Nishida N, Harris DA, Vilette D, Laude H, Frobert Y (2000). Successful transmission of three mouse-adapted scrapie strains to murine neuroblastoma cell lines overexpressing wild-type mouse prion protein.. J Virol.

[30] Bosque PJ, Prusiner SB (2000). Cultured cell sublines highly susceptible to prion infection.. J Virol.

[31] Vorberg I, Raines A, Story B, Priola SA (2004). Susceptibility of common fibroblast cell lines to transmissible spongiform encephalopathy agents.. J Infect Dis.

[32] Lehmann S (2005). Prion propagation in cell culture.. Methods Mol Biol.

[33] Mahal SP, Baker CA, Demczyk CA, Smith EW, Julius C (2007). Prion strain discrimination in cell culture: the cell panel assay.. Proc Natl Acad Sci U S A.

[34] Gajdusek DC (1988). Transmissible and non-transmissible amyloidoses: Autocatalytic post-translational conversion of host precursor proteins to ß-pleated configurations.. J Neuroimmunology.

[35] Jarrett JT, Lansbury PJ (1993). Seeding “one-dimensional crystallization” of amyloid: a pathogenic mechanism in Alzheimer's disease and scrapie?. Cell.

[36] Come JH, Fraser PE, Lansbury PJ (1993). A kinetic model for amyloid formation in the prion diseases: importance of seeding.. Proc Natl Acad Sci U S A.

[37] Weissmann C (2004). The state of the prion.. Nat Rev Microbiol.

[38] Weissmann C (2005). Birth of a prion: spontaneous generation revisited.. Cell.

[39] Collinge J, Clarke AR (2007). A general model of prion strains and their pathogenicity.. Science.

[40] Kuwata K, Kamatari YO, Akasaka K, James TL (2004). Slow conformational dynamics in the hamster prion protein.. Biochemistry.

[41] O'Sullivan DB, Jones CE, Abdelraheim SR, Brazier MW, Toms H (2009). Dynamics of a truncated prion protein, PrP(113–231), from (15)N NMR relaxation: order parameters calculated and slow conformational fluctuations localized to a distinct region.. Protein Sci.

[42] Younan ND, Klewpatinond M, Davies P, Ruban AV, Brown DR (2011). Copper(II)-induced secondary structure changes and reduced folding stability of the prion protein.. J Mol Biol.

[43] Weissmann C (1991). A “unified theory” of prion propagation.. Nature.

[44] Wang F, Wang X, Yuan CG, Ma J (2010). Generating a prion with bacterially expressed recombinant prion protein.. Science.

[45] Nandi PK (1998). Polymerization of human prion peptide HuPrP 106–126 to amyloid in nucleic acid solution.. Arch Virol.

[46] Gabus C, Derrington E, Leblanc P, Chnaiderman J, Dormont D (2001). The prion protein has RNA binding and chaperoning properties characteristic of nucleocapsid protein NCP7 of HIV-1.. J Biol Chem.

[47] Gomes MP, Millen TA, Ferreira PS, e Silva NL, Vieira TC (2008). Prion protein complexed to N2a cellular RNAs through its N-terminal domain forms aggregates and is toxic to murine neuroblastoma cells.. J Biol Chem.

[48] Nandi PK (2008). Conformational changes of prion protein and nucleic acid arising from their interaction and relation of the altered structures in causing prion disease.. Mini Rev Med Chem.

[49] Deleault NR, Lucassen RW, Supattapone S (2003). RNA molecules stimulate prion protein conversion.. Nature.

[50] Deleault NR, Harris BT, Rees JR, Supattapone S (2007). Formation of native prions from minimal components in vitro.. Proc Natl Acad Sci U S A.

[51] Wang F, Zhang Z, Wang X, Li J, Zha L (2011). Genetic informational RNA is not required for recombinant prion infectivity.. J Virol.

[52] Kuczius T, Koch R, Keyvani K, Karch H, Grassi J (2007). Regional and phenotype heterogeneity of cellular prion proteins in the human brain.. Eur J Neurosci.

[53] DeArmond SJ, Sanchez H, Yehiely F, Qiu Y, Ninchak-Casey A (1997). Selective neuronal targeting in prion disease.. Neuron.

[54] Telling GC, Scott M, Mastrianni J, Gabizon R, Torchia M (1995). Prion propagation in mice expressing human and chimeric PrP transgenes implicates the interaction of cellular PrP with another protein.. Cell.

[55] Nishina KA, Deleault NR, Mahal SP, Baskakov I, Luhrs T (2006). The stoichiometry of host PrPC glycoforms modulates the efficiency of PrPSc formation in vitro.. Biochemistry.

[56] Salamat MK, Dron M, Chapuis J, Langevin C, Laude H (2011). Prion propagation in cells expressing PrP glycosylation mutants.. J Virol.

[57] Cancellotti E, Bradford BM, Tuzi NL, Hickey RD, Brown D (2010). Glycosylation of PrPC determines timing of neuroinvasion and targeting in the brain following transmissible spongiform encephalopathy infection by a peripheral route.. J Virol.

[58] Neuendorf E, Weber A, Saalmueller A, Schatzl H, Reifenberg K (2004). Glycosylation deficiency at either one of the two glycan attachment sites of cellular prion protein preserves susceptibility to bovine spongiform encephalopathy and scrapie infections.. J Biol Chem.

[59] Angers RC, Kang HE, Napier D, Browning S, Seward T (2010). Prion strain mutation determined by prion protein conformational compatibility and primary structure.. Science.

[60] Chesebro B, Trifilo M, Race R, Meade-White K, Teng C (2005). Anchorless prion protein results in infectious amyloid disease without clinical scrapie.. Science.

[61] McNally KL, Ward AE, Priola SA (2009). Cells expressing anchorless prion protein are resistant to scrapie infection.. J Virol.

[62] Lee AM, Paulsson JF, Cruite J, Andaya AA, Trifilo MJ (2011). Extraneural manifestations of prion infection in GPI-anchorless transgenic mice.. Virology.

[63] Chesebro B, Race B, Meade-White K, Lacasse R, Race R (2010). Fatal transmissible amyloid encephalopathy: a new type of prion disease associated with lack of prion protein membrane anchoring.. PLoS Pathog.

[64] Campana V, Caputo A, Sarnataro D, Paladino S, Tivodar S (2007). Characterization of the properties and trafficking of an anchorless form of the prion protein.. J Biol Chem.

[65] Priola SA, McNally KL (2009). The role of the prion protein membrane anchor in prion infection.. Prion.

[66] Dickinson AG (1976). Scrapie in sheep and goats.. Front Biol.

[67] Groschup MH, Gretzschel A, Kuczius T, Hornlimann B, Riesner D, Kretschmar H (2006). Prion strains.. Prions in humans and animals.

[68] Bruce ME, McConnell I, Fraser H, Dickinson AG (1991). The disease characteristics of different strains of scrapie in Sinc congenic mouse lines: implications for the nature of the agent and host control of pathogenesis.. J Gen Virol.

[69] Nishina KA, Supattapone S (2007). Immunodetection of glycophosphatidylinositol-anchored proteins following treatment with phospholipase C.. Anal Biochem.

[70] Tulsiani DR, Harris TM, Touster O (1982). Swainsonine inhibits the biosynthesis of complex glycoproteins by inhibition of Golgi mannosidase II.. J Biol Chem.

[71] Tulsiani DR, Touster O (1983). Swainsonine causes the production of hybrid glycoproteins by human skin fibroblasts and rat liver Golgi preparations.. J Biol Chem.

[72] Crispin M, Aricescu AR, Chang VT, Jones EY, Stuart DI (2007). Disruption of alpha-mannosidase processing induces non-canonical hybrid-type glycosylation.. FEBS Lett.

[73] Browning S, Baker CA, Smith E, Mahal SP, Herva ME (2011). Abrogation of complex glycosylation by swainsonine results in strain- and cell-specific inhibition of prion replication.. J Biol Chem.

[74] Li J, Browning S, Mahal SP, Oelschlegel AM, Weissmann C (2010). Darwinian evolution of prions in cell culture.. Science.

[75] Weissmann C, Li J, Mahal SP, Browning S (2011). Prions on the move.. EMBO Rep.

[76] Li J, Mahal SP, Demczyk CA, Weissmann C (2011). Mutability of prions.. EMBO Rep.

[77] Oelschlegel AM, Fallahi M, Ortiz-Umpierre S, Weissmann C (2012). The Extended Cell Panel Assay (ECPA) characterizes the relationship of RML, 79A and 139A prion strains and reveals conversion of 139A to 79A-like prions in cell culture.. J Virol.

[78] Kimberlin RH, Cole S, Walker CA (1987). Temporary and permanent modifications to a single strain of mouse scrapie on transmission to rats and hamsters.. J Gen Virol.

[79] Bruce ME (2003). TSE strain variation.. Br Med Bull.

[80] Legname G, Nguyen HO, Peretz D, Cohen FE, DeArmond SJ (2006). Continuum of prion protein structures enciphers a multitude of prion isolate-specified phenotypes.. Proc Natl Acad Sci U S A.

[81] Colby DW, Prusiner SB (2011). De novo generation of prion strains.. Nat Rev Microbiol.

[82] Mahal SP, Browning S, Li J, Suponitsky-Kroyter I, Weissmann C (2010). Transfer of a prion strain to different hosts leads to emergence of strain variants.. Proc Natl Acad Sci U S A.

[83] Williamson RA, Peretz D, Pinilla C, Ball H, Bastidas RB (1998). Mapping the prion protein using recombinant antibodies.. J Virol.

[84] Saa P, Castilla J, Soto C (2005). Cyclic amplification of protein misfolding and aggregation.. Methods Mol Biol.

