# Supplementary figures and images for: Propagation of RML Prions in Mice Expressing PrP Devoid of GPI Anchor Leads to Formation of a Novel, Stable Prion Strain
Source: PLoS Pathog. 2012 Jun 7;8(6):e1002746. doi: 10.1371/journal.ppat.1002746 (PMC3369955; doi:10.1371/journal.ppat.1002746)

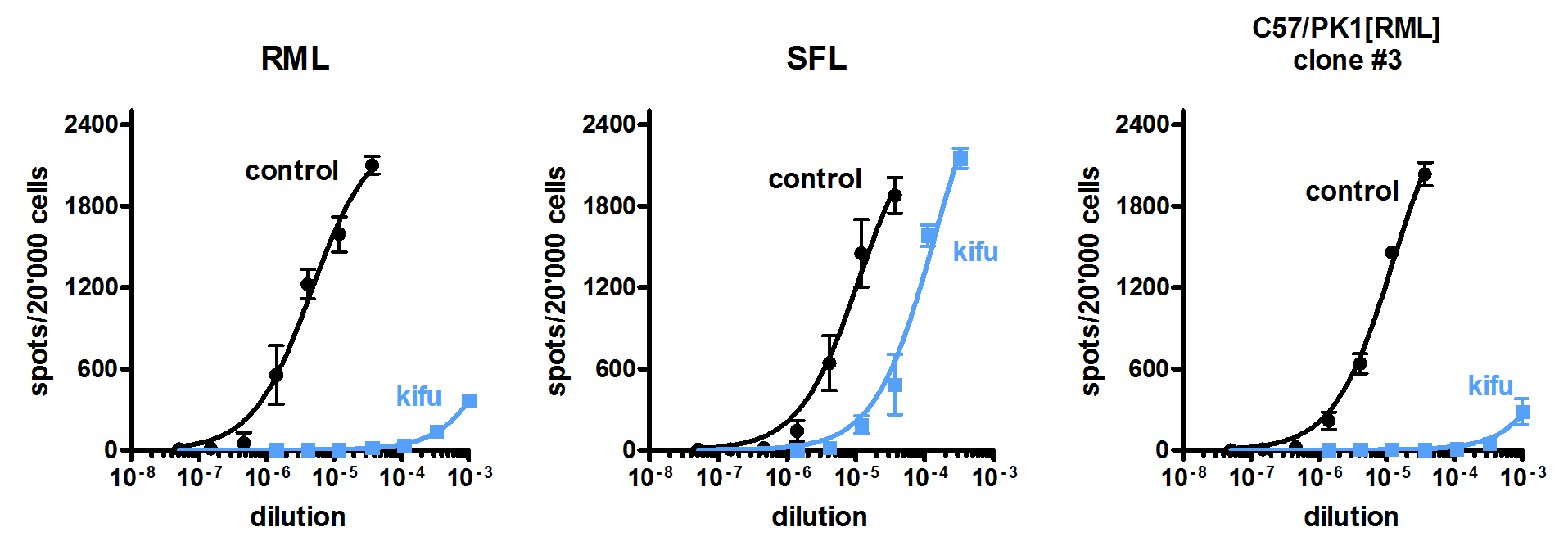

Supplement: Figure S1 — Determination of kifunensine-susceptibility of twelve independent clones of RML. PK1 cells were infected with a high dilution of RML prions as described in the Methods section. Prions from RML-infected clones were inoculated in C57BL/6 mice and serial dilutions of homogenized brains were analyzed by the SSCA on PK1 cells in the presence or absence of 5 µg kifu/ml. All 12 clones were indistinguishable from the original RML in being strongly inhibited by kifu. RML and the clones were distinctly different from SFL, which was only weakly inhibited by kifu. The SSCA of only one representative of the 12 clones (#3) is shown. (TIF) [file ppat.1002746.s001.tif]

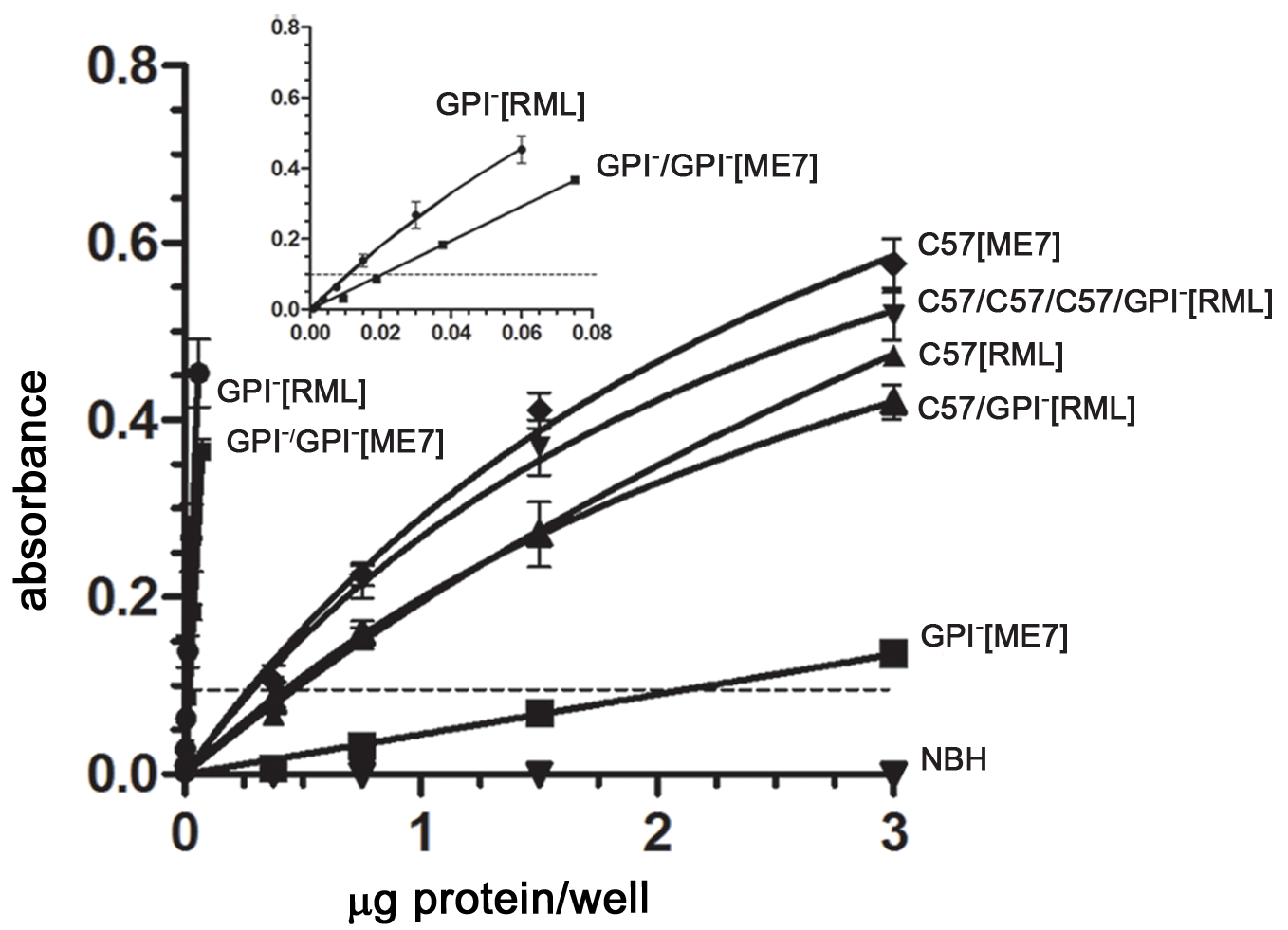

Supplement: Figure S2 — Sandwich ELISA of samples from Figure 1A . Absorbance of quadruplicate samples is plotted against input protein on a linear plot, to show that absorbance is almost linear with input protein up to1.5 µg/well. NBH, uninfected C57 brain homogenate. (TIF) [file ppat.1002746.s002.tif]

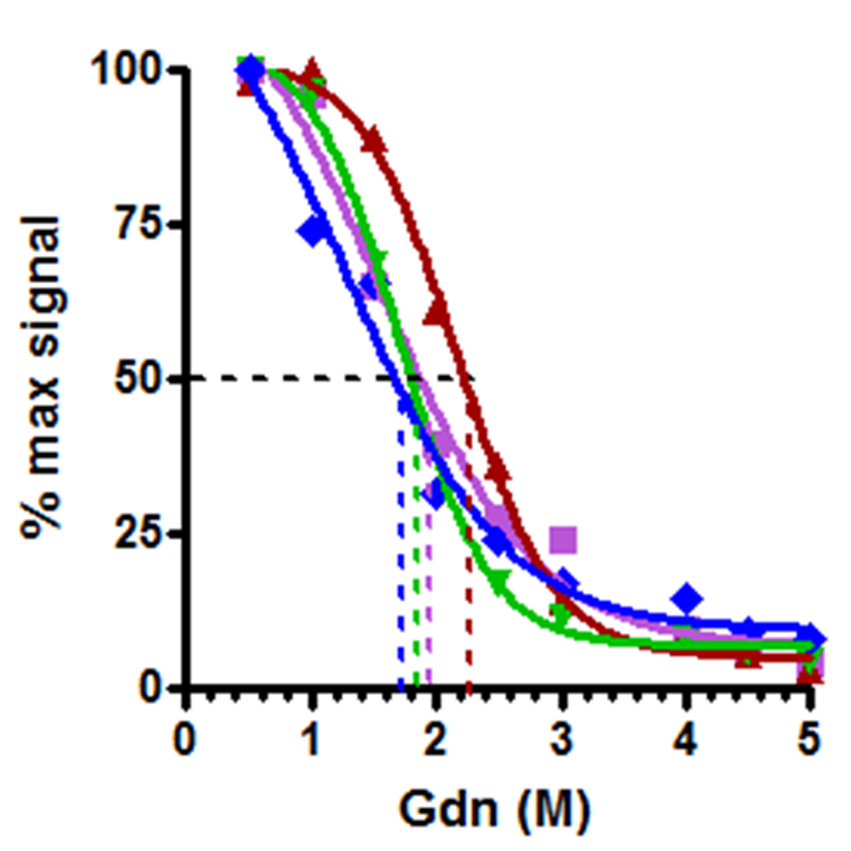

Supplement: Figure S3 — Conformational stability of PrPres from various strains. The assay was described in the Methods section. The highest value for each curve was set to 100% and the concentration of GndCl at which 50% of the PrPres was digested by PK is represented by the dotted lines. PrPres from RML (lilac), C57/GPI−[RML] (green) and C57/C57/GPI−[RML] (blue) showed no significant differences in stabilities, which ranged from 1.7 to 1.9 M GndCl. GPI−[RML] (red) had a marginally higher stability (2.2 M GndCl). (TIF) [file ppat.1002746.s003.tif]

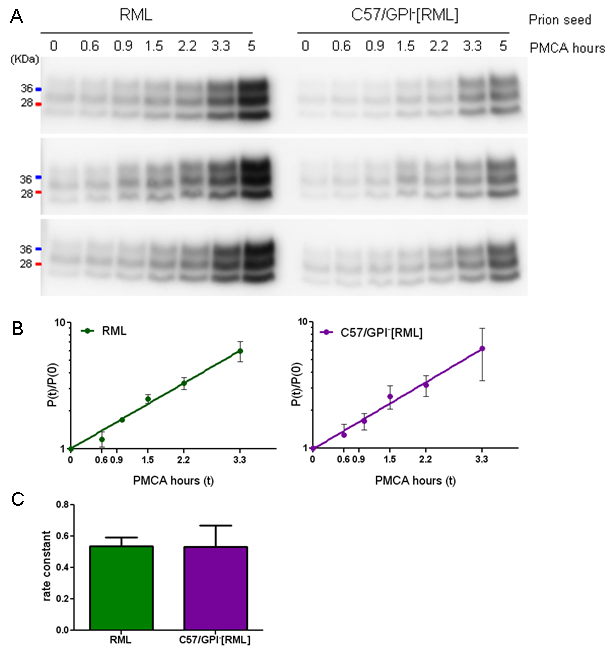

Supplement: Figure S4 — Kinetic Protein Misfolding Cyclic Amplification (PMCA). PMCA was performed to identify differences in rate constants of amplification of PrPres from C57[RML] and C57/GPI−[RML] brains. PMCA substrate (uninfected C57 brain homogenate) was prepared as described [84], but without centrifugation. Prion “seeds”, RML and SFL (C57/GPI−[RML]) PrPres were adjusted to the same levels, as determined by densitometric analysis on western blots. (A) PMCA reaction mixtures contained 441 µl substrate and either 9 µl of a 10−2 dilution of RML or an equivalent amount of SFL PrPres. PMCA was performed with 60-µl aliquots of the reaction mixtures dispensed in triplicate into 200-µl PCR tubes (Axygen) containing 37±3 mg of 1.0 mm Zirconia/Silica beads (Biospec products). Samples were subjected to cycles of 20 second sonication and 30 min incubation at 37°C for 0, 0.6, 0.9, 1.5, 2.2, 3.3 or 5 h, using a Misonix 3000 sonicator at power setting 8.5. To measure amplified PrPres, 20-µl aliquots were PK-digested and 10 µl were electrophoresed and analyzed as previously described [73]. (B) Western blots were quantified and the ratio rPrPres(time t)/rPrPres(time 0) was plotted on a log scale against time. The rate constants were calculated from the logarithmic phases of the kinetics (up to 3.3 h). (C) There were no statistically significant differences (t student) in the rate constants of RML and C57/GPI−[RML] PrPres amplification. (TIF) [file ppat.1002746.s004.tif]
