# Supplementary material for: Propagation of RML Prions in Mice Expressing PrP Devoid of GPI Anchor Leads to Formation of a Novel, Stable Prion Strain
Source: PLoS Pathog. 2012 Jun 7;8(6):e1002746. doi: 10.1371/journal.ppat.1002746 (PMC3369955; doi:10.1371/journal.ppat.1002746)
Supplement: Table S1 — Incubation periods and clinical signs for C57BL/6 and tgGPI− mice inoculated with various inocula. (DOCX) [file ppat.1002746.s005.docx]

**Table S1. Incubation periods and clinical signs for C57BL/6 and tgGPI^-^ mice inoculated with various inocula.**

| **#** | **Inoculum** | **Mouse** | **Culled (dpi)** | **Signs at day of euthanasia** |
| --- | --- | --- | --- | --- |
| **1*** | CD1[RML] | C57 | 142 ±12  n=29 | clinically sick, stopped nesting, lateral deviation of hind limbs. Not terminal when culled because of vaginal lesions due to urinary incontinence in some mice (AMO, SO) |
| **2a*** | CD1[RML] | GPI^-^ | 301 ±0 **†**  n=2 | mildly ataxic (AS) |
| 2b | CD1[RML] | GPI^-^ | 307 ±0 **†**  n=2 | neurological signs in 1 of 2 mice (AS) |
| **2c*** | CD1[RML] | GPI^-^ | 342 ±8  n=5/7 | scruffy, hunched, circling, lateral deviation of hind limbs, ataxic, weak hind limbs, frantic eating, compulsive grooming, dragging body, legs spread to sides, no foot correction, tremor, breathing heavily, weight loss, squinting; 2 mice alive, but clinical at 449 days (as of 2/8/2012) (AMO, SO) |
| 3a* | GPI^-^[RML] | GPI^-^ | 196 ±0 **†**  n=3 | clinical from 168 dpi (AS) |
| 3b | GPI^-^[RML] | GPI^-^ | 263 ±47  n=8 | scruffy, hunched, lateral deviation of hind limbs, ataxic, wobbling, poor balance, dragging hind limbs, depressed expression, unconscious scratching, squinting, moving with eyes closed, heavy fast breathing, weight loss, not eating, dehydrated, hyperactive to inactive in 24 h (AMO, SO) |
| **4a*** | GPI^-^[RML] | C57 | 138 ±0 **†** n=4 | clinical (AS) |
| **4b*** | GPI^-^[RML] | C57 | 150 ±9  n=3 | scruffy, hunched, circling, lateral deviation of hind limbs, dragging hind limbs, ataxic, urinary incontinence, huge bladder, lethargic, squinting dehydrated, little movement, weight loss (AMO, SO) |
| 4c | GPI^-^[RML] | C57 | 168  n=1 | scruffy, lateral deviation of hind limbs, ataxic, falling over, squinting, weight loss, not eating, dehydrated (AMO, SO) |
| **5a*** | C57/GPI^-^[RML] | C57 | 153 ±11  n=4 | scruffy, hunched, head tilt, myoclonus, shivering, ataxic, tail plasticity, weight loss, lethargic, decreased respiration (AMO, SO) |
| **5b*** | C57/GPI^-^[RML] | C57 | 146 ±5  n=2 | scruffy, hunched, lateral deviation of hind limbs, ataxic, weak hind limbs, large bladder, dehydrated, weight loss (AMO, SO) |
| **6*** | C57/C57/GPI^-^[RML] | C57 | 154 ±11  n=6 | scruffy, hunched, lateral deviation of hind limbs, weak hind limbs, dragging rear on ground, ataxic, large bladder, urinary incontinence, not alert, not moving, squinting, eyes closed, dehydrated, not eating, drinking or defecating, shivering (AMO, SO) |
| **7a*** | C57[79A] | C57 | 133 ±1  n=19 | restless, uncoordinated when agitated, ataxia, urinary incontinence (AS) |
| 7b | C57[79A] | C57 | 146 ±3  n=3 | hunched, lateral deviation of hind limbs, dragging rear, myoclonus, leaning to the right, large bladder, urinary incontinence, squinting (AMO, SO) |
| **8a*** | C57[79A] | GPI^-^ | 295 ±0  n=2 | clinical, lethargic (AS) |
| 8b | C57[79A] | GPI^-^ | 243  n=1 | no notes; other found dead at 241 dpi (AS) |
| 8c | C57[79A] | GPI^-^ | >368  n=5/8 | scruffy, hunched, alert but tranquil, lateral deviation of hind limbs, difficulty moving, leaning to one side, urinary incontinence, squinting, weight loss, dehydrated, inactive, unresponsive. [*3 alive as of 2/9/2012*] (AMO, SO) |
| 9 | GPI^-^[79A] | GPI^-^ | 291 ±54  n=8 | scruffy, hunched, not alert, but restless to hyperactive, lateral deviation of hind limbs, ataxic, leaning to one side, circling, weight loss squinting, dehydrated, fast respiration (AMO, SO) |
| 10 | GPI^-^[79A] | C57 | 155 ±6  n=3 | scruffy, hunched, not active or hyperactive, lateral deviation of hind limbs, ataxic, balance impaired, squinting, weight loss (AMO, SO) |
| **11a*** | C57[139A] | C57 | 232  n=1 | severe neurological signs in 1 of 2; other found dead at 190 dpi (AS) |
| **11b*** | C57[139A] | C57 | 132 ±7 **†**  n=11 | clinically sick, hunched, lethargic (AS) |
| 11c | C57[139A] | C57 | 156 ±2  n=4 | hunched, scruffy, lateral deviation of hind limbs, weak hind limbs, urinary incontinence, large bladder (AMO, SO) |
| **12a*** | C57[139A] | GPI^-^ | 215 ±0 **†**  n=5 | head tilt, mildly neurological (AS) |
| 12b | C57[139A] | GPI^-^ | 279 ±27  n=8 | scruffy, hunched, hyperactive, circling, lateral deviation, ataxic balance impaired, squinting, , weight loss, dehydrated (AMO, SO) |
| 13 | GPI^-^[139A] | GPI^-^ | 224 ±42  n=7 | scruffy, hunched, inactive, lethargic, alert when stimulated, lateral deviation, ataxic, circling, dragging body, unable to move, splayed hind limbs, weight loss, squinting, dehydrated, abnormal respiration (AMO, SO) |
| 14a | C57[22L] | C57 | 145 ±3  n=19 | ruffled coat, tilting to the right, “sailor walk”, thin (AS) |
| 14b | C57[22L] | C57 | 141 ±6  n=8 | scruffy, hunched, hyperactive, loss of alertness in some, lateral deviation of hind limbs, hind limb weakness, ataxic, balance impaired, squinting, severe weight loss, dehydrated (AMO, SO) |
| **14c*** | C57[22L] | C57 | 149 ±5  n=20 | hunched, scruffy, hyperactive, loss of alertness & activity in some, lateral deviation of hind limbs, slow walking, ataxia, balance impaired, squinting, severe weight loss, not eating (AMO, SO) |
| **15a*** | C57[22L] | GPI^-^ | 301 ±0 **†**  n=2 | no notes (AS) |
| 15b | C57[22L] | GPI^-^ | 307 ±0 **†**  n=2 | both ataxic (AS) |
| 16 | GPI^-^ [22L] | GPI^-^ | 227 ±0 **†**  n=4 | neurological signs (AS) |
| 17 | GPI^-^ [22L] | C57 | 143 ±0 **†**  n=4 | neurological signs (AS) |
| 18a | C57[ME7] | C57 | 140 ±5  n=12 | uncoordinated, limping, started showing clinical signs at 130 dpi (AS) |
| **18b*** | C57[ME7] | C57 | 138 ±4  n=11 | abnormal placement of hind limbs, incontinence, weight loss, ataxia (AS) |
| **19a*** | C57[ME7] | GPI^-^ | 301 ±0 **†**  n=2 | no notes (AS) |
| **19b*** | C57[ME7] | GPI^-^ | 307 ±0 **†**  n=2 | some very thin (AS) |
| **20*** | GPI^-^ [ME7] | C57 | 170 ±0  n=5 | clinical from 121 dpi (AS) |
| **21*** | GPI^-^ [ME7] | GPI^-^ | 447 ±64 **†**  n=4 | 1 clinical, 3 of 4 culled due to eye infections/bladder problems (AS) |
| 22 | GPI^-^/GPI^-^[ME7] | C57 | [>160 ±]  n=4 | ongoing (AMO, SO) |

*, brains used in assays

**†**, culled before reaching terminal stage

Most prion-inoculated tgGPI^-^ mice show increased appetite during the clinical phase. (AMO, SO) and (AS) assessed mice independently in successive periods
