# Supplementary material for: Propagation of RML Prions in Mice Expressing PrP Devoid of GPI Anchor Leads to Formation of a Novel, Stable Prion Strain
Source: PLoS Pathog. 2012 Jun 7;8(6):e1002746. doi: 10.1371/journal.ppat.1002746 (PMC3369955; doi:10.1371/journal.ppat.1002746)
Supplement: Text S1 — Incubation periods, clinical signs, biochemical and infectivity analyses of prion strains in tgGPI- and wild-type C57BL/6 mice. (DOCX) [file ppat.1002746.s006.docx]

**SUPPORTING TEXT**

**Incubation periods, clinical signs, biochemical and infectivity analyses of prion strains in tgGPI- and wild-type C57BL/6 mice**

**Disease symptoms** were milder and appeared later, if at all, in tgGPI^-^ mice than in wild-type C57BL/6 mice (Table S1).

**Presence of SFL-like prions in RML populations** was assessed by cloning RML in PK1 cells and inoculating prions from 12 clones into C57BL/6 mice. Dilution series of terminally sick brains were assayed on PK1 cells in the presence or absence of kifunensine (kifu). All 12 clones of RML showed a distinctly different susceptibility to kifunensine than SFL (Figure S1), showing that SFL was not a major component of RML populations.

**The PrP^res^ content of prion-infected GPI^-^ or C57 brain homogenates** was assayed by sandwich ELISA and absorbance was plotted against log[input protein] (Figure S2). The abundance of a sample relative to that of RML can be read off by comparing the amounts of protein required to give the same absorbance.

**The conformational stability** of PrP^Sc^ from “authentic” brain[RML] could not be distinguished from that from C57/GPI^-^[RML] or C57/C57/GPI^-^[RML] (which harbor the “SFL strain”) (Figure S3). C57/[RML] and C57/GPI^-^[RML] were equally efficient seeds for brain-homogenate based PMCA (Figure S4).
